# Challenges and strategies for implementing genomic services in diverse settings: experiences from the Implementing GeNomics In pracTicE (IGNITE) network

**DOI:** 10.1186/s12920-017-0273-2

**Published:** 2017-05-22

**Authors:** Nina R. Sperber, Janet S. Carpenter, Larisa H. Cavallari, Laura J. Damschroder, Rhonda M. Cooper-DeHoff, Joshua C. Denny, Geoffrey S. Ginsburg, Yue Guan, Carol R. Horowitz, Kenneth D. Levy, Mia A. Levy, Ebony B. Madden, Michael E. Matheny, Toni I. Pollin, Victoria M. Pratt, Marc Rosenman, Corrine I. Voils, Kristen W. Weitzel, Russell A. Wilke, R. Ryanne Wu, Lori A. Orlando

**Affiliations:** 1 Division of General Internal Medicine, Duke University School of Medicine, Durham, NC USA; 2 Indiana University School of Nursing, Indianapolis, IN USA; 3 Department of Pharmacotherapy and Translational Research and Center for Pharmacogenomics, University of Florida, Gainesville, FL USA; 4 Implementation Pathways, LLC and VA Ann Arbor Center for Clinical Management Research, Ann Arbor, USA; 5 University of Florida, College of Pharmacy and Medicine and Center for Pharmacogenomics, Gainesville, USA; 6 Vanderbilt University Medical Center, Nashville, USA; 7 Duke Center for Applied Genomics & Precision Medicine, Duke University, Durham, NC USA; 8 University of Maryland School of Medicine, Baltimore, USA; 9 Icahn School of Medicine at Mount Sinai, New York, USA; 10 National Human Genome Research Institute (NHGRI), Rockville, USA; 11 Vanderbilt University Medical Center, Tennessee Valley HealthCare System VA, Nashville, USA; 12 William S. Middleton Memorial Veterans Hospital, Madison, WI USA; 13 Department of Surgery, University of Wisconsin-Madison, Madison, WI USA; 14 Sanford School of Medicine, University of South Dakota, Vermillion, USA; 15 Duke University, Duke-National University of Singapore Medical School, 8 College Road, Singapore, 169857 Singapore; 16 VA Health Services Research & Development, Durham VA Health Care System, 411 West Chapel Hill Street, Suite 600, Durham, NC 27701 USA

**Keywords:** Precision medicine, Pharmacogenomics, Electronic health record, Patient engagement, Provider engagement, Implementation

## Abstract

**Background:**

To realize potential public health benefits from genetic and genomic innovations, understanding how best to implement the innovations into clinical care is important. The objective of this study was to synthesize data on challenges identified by six diverse projects that are part of a National Human Genome Research Institute (NHGRI)-funded network focused on implementing genomics into practice and strategies to overcome these challenges.

**Methods:**

We used a multiple-case study approach with each project considered as a case and qualitative methods to elicit and describe themes related to implementation challenges and strategies. We describe challenges and strategies in an implementation framework and typology to enable consistent definitions and cross-case comparisons. Strategies were linked to challenges based on expert review and shared themes.

**Results:**

Three challenges were identified by all six projects, and strategies to address these challenges varied across the projects. One common challenge was to increase the relative priority of integrating genomics within the health system electronic health record (EHR). Four projects used data warehousing techniques to accomplish the integration. The second common challenge was to strengthen clinicians’ knowledge and beliefs about genomic medicine. To overcome this challenge, all projects developed educational materials and conducted meetings and outreach focused on genomic education for clinicians. The third challenge was engaging patients in the genomic medicine projects. Strategies to overcome this challenge included use of mass media to spread the word, actively involving patients in implementation (e.g., a patient advisory board), and preparing patients to be active participants in their healthcare decisions.

**Conclusions:**

This is the first collaborative evaluation focusing on the description of genomic medicine innovations implemented in multiple real-world clinical settings. Findings suggest that strategies to facilitate integration of genomic data within existing EHRs and educate stakeholders about the value of genomic services are considered important for effective implementation. Future work could build on these findings to evaluate which strategies are optimal under what conditions. This information will be useful for guiding translation of discoveries to clinical care, which, in turn, can provide data to inform continual improvement of genomic innovations and their applications.

## Background

Precision medicine, which addresses individual variability in effectiveness of disease prevention and treatment strategies, is a rapidly growing field, with application on a broad scale spurred by innovations resulting from the accumulation of massive amounts of health and biologic data. The Precision Medicine Initiative, launched by President Obama in 2015, reflects a public health commitment to developing not only technologies but also infrastructure for harnessing and sharing data from a national initial cohort of one million volunteers to develop precision medicine applications [1, 2]. Discovery is proceeding rapidly, and there is a need for systems capable of integrating genetic evidence-based applications into routine health care [3].

Even with agreed-upon clinically actionable recommendations, uptake of recommendations by clinicians and health systems is not optimal. For example, though there is adequate evidence that testing for Lynch Syndrome, the most common form of hereditary colorectal cancer, could improve outcomes for patients and relatives, there has been a low level of screening in high-risk patients [4]. Similarly, despite sufficient evidence of certain gene/drug interactions such as between *CYP2C19* and clopidogrel and *CYP2C9/VKORC1* and warfarin, routine genotyping is not performed either preemptively or at the time of prescribing [5, 6].

In order to optimize potential public health benefits from genetic and genomic innovations, there is a need to focus on developing both the evidence base for their impact on health outcomes and also strategies that can be used to put them into practice at a broad scale. Implementation science, the systematic study of strategies to adopt and integrate evidence-based interventions in real-world settings seeks to shed light on how to accelerate adoption of genomic innovations. Understanding the influences of contextual factors on implementation strategies and adaptations of genomic interventions is essential [7–9].

From previous work completed in single settings, it is known that barriers to adoption of genomic medicine include unclear organizational policies or criteria for use, inconsistent modes for integrating genomic information into the electronic health record system, and concerns about cost of testing for patients and institutions. At an individual level, factors related to poor uptake include lack of knowledge by physicians on how to interpret information and lack of understanding by patients about genomic testing and the implication of results [8, 10]. These individual and system level barriers can interact such that decision makers may not consider genomics at all or may consider the obstacles too great in planning new models of care [11]. Johnson and Weitzel assert that, in private practice settings, local champions who can advocate for the value of genomic medicine are essential to advance implementation [12].

Although there is a body of literature describing challenges to routine use of genomic innovation in specific settings, widespread integration of genomic innovations into clinical care, a public health objective [13], could be advanced by collaborative identification of best practices based on experiences across settings [3]. We present common strategies for implementation of genomic innovations across six diverse projects (Table 1) within the National Human Genome Research Institute (NHGRI)-funded network called IGNITE (Implementing GeNomics In pracTicE) [14]. Our results are organized using a well-accepted implementation framework, the Consolidated Framework for Implementation Research (CFIR), to systematically define barriers and facilitators [15–17].

**Table 1:** IGNITE (Implementing GeNomics In pracTicE) projects

IGNITE Site	Principal Investigators	Study Name	Study Goals
Duke University School of Medicine	Geoffery Ginsburg, MD, PhD and Lori A. Orlando, MD, MHS	Implementation, Adoption, and Utility of Family History in Diverse Care Settings	• Develop an optimal strategy for implementing a patient-facing web-based family health history tool, MeTree, into routine clinical practice in diverse settings. • Demonstrate the effectiveness of MeTree in increasing uptake of risk stratified evidence-based prevention guidelines. • Create a standardized family health history storage database that can integrate with electronic medical records for bi-directional communication of family and personal history data and risk assessment results.
Indiana University School of Medicine	Paul Dexter, MD and Todd Skaar, PhD	INGENIOUS: Indiana GENomics Implementation, an Opportunity for the Under-Served	• Test the hypothesis that a CLIA certified genotyping targeted at 24 widely used drugs is associated with significant reductions in hospital and outpatient economic costs incurred over 1 year. • Test whether pharmacogenetic testing is associated with significant improvements in clinical outcomes over 1 year.
Icahn School of Medicine at Mount Sinai	Carol R. Horowitz, MD, MPH	The GUARDD Study: Genetic testing to Understand and Address Renal Disease Disparities	• Conduct a randomized trial in a network of community health centers and primary care facilities to study processes, effects and challenges of incorporating information for apolipoprotein L1 (APOL1)-attributable genetic risk for end stage kidney disease in patients of African ancestry with hypertension.
University of Florida College of Pharmacy	Julie A. Johnson, PharmD	Genomic Medicine Implementation: The Personalized Medicine Program (PMP)	• Partner with health professionals and patients at UF Health and across the state to develop, implement, study and refine methods that allow genetic information to be used as a routine part of patient care. • Engage in expansion to Private and Community-based Practices in Florida. • Develop programs in Genomic Medicine Education.
University of Maryland School of Medicine	Toni I. Pollin, MS, PhD	Genomic Diagnosis and Individualized Therapy of Highly Penetrant Genetic Diabetes	• Implement and evaluate a Personalized Diabetes Medicine Program (PDMP) in four diverse health care settings to enhance the identification and opportunities for individualized therapy of individuals and family members affected by highly penetrant/ monogenic diabetes.
Vanderbilt University Medical Center	Joshua C. Denny, MD, MS and Mia Levy, MD, PhD	Integrated, Individualized, and Intelligent Prescribing (I^3^P) Network	• Develop, maintain, and disseminate genome-informed clinical decision support within Adopter Sites. • Select and prospectively genotype patients within Adopter Sites for subsequent genotype-tailored therapy. • Evaluate the impact of integrating genetic data and clinical decision support into Adopter Site EMRs.

*CLIA* Clinical Laboratory Improvement Amendments

## Methods

We applied a multiple case study approach with each IGNITE project considered as a case, or unit of analysis. Some projects were implemented in more than one health care setting and thus, for those cases, the challenges and strategies were aggregated to explain implementation across different health care settings at the project level. This case series sought to address research questions related to understanding how genomic medicine innovations are implemented in real-world clinical settings, using in-depth information from within each case and additional insights by comparing across cases [18].

One implementation researcher (NS) elicited data about strategies used by disseminating a form, consisting of an electronic spreadsheet, to projects, with strategies defined and grouped *a priori* according to a taxonomy published by the Expert Recommendations for Implementing Change (ERIC) project [19]. (For example, strategies related to adapting and tailoring context were listed on one worksheet and strategies related to training and educating stakeholders were listed in another worksheet.) Project leaders responded via email as to whether and how they had used each strategy. The researcher conducted an independent review to check that strategies described fit with the definitions established by the ERIC project and identify strategies that were used by at least three projects.

The final step involved organizing strategies according to relevant challenges that had been identified by all projects in an open-ended discussion during an in-person meeting, 21 months after the launch of IGNITE. Two implementation researchers (LD, NS) mapped these challenges to constructs from the CFIR, resolved slight disagreements through discussion, and achieved 100% consensus on the mapping: the CFIR, designed to provide an overarching typology to promote implementation theory development about determinants of implementation across multiple contexts, has been widely used by implementation scientists to guide data collection, analysis or reporting, demonstrating that it can be used to generalize information about implementation experiences [17]. Strategies presented below are those that relate to one of these three common challenges via shared themes (e.g., the electronic health record (EHR) was identified as a locus for both challenge and strategy). Documentation of the challenges and strategies was verified via email and telephone conference discussion with investigators from each project, and the relationships between challenges and strategies were validated by an implementation expert (LD).

## Results

Three challenges were identified by all six projects: 1) prioritizing integration of genomics into the EHR, 2) improving clinicians’ knowledge and beliefs about genomic medicine, and 3) engaging patients to participate in the genomic medicine projects. Below, we describe the nature of these common challenges and strategies used by projects to address them. Table 2 provides an overview of these results.

**Table 2:** Implementation strategies by challenge and IGNITE project

Implementation Challenge	Duke	IU	Mount Sinai	UF	UM	Vanderbilt
Implementation Strategy						
Prioritizing integration of genomics into EHR						
Use data warehousing techniques		✓	✓		✓	✓
Improving clinician knowledge and beliefs about genomic medicine						
Develop educational materials for clinicians	✓	✓	✓	✓	✓	✓
Conduct educational meetings with clinicians		✓	✓	✓	✓	✓
Conduct educational outreach in clinical settings		✓		✓	✓	✓
Engaging patients to participate in genomic medicine projects						
Use mass media to educate potential users	✓	✓	✓	✓	✓	✓
Involve patients in implementation activities		✓	✓		✓	
Prepare patients to be active participants	✓		✓		✓	✓

*IGNITE* Implementing GeNomics in PracTicE, *IU* Indiana University School of Medicine, *Mount Sinai* Icahn School of Medicine at Mount Sinai, *UF* University of Florida College of Pharmacy, *UM* University of Maryland School of Medicine, *Vanderbilt* Vanderbilt University Medical Center, *EHR* Electronic Health Record

### Challenge 1: prioritizing integration of genomics into EHR

The more that an innovation is perceived and promoted as a priority from within an organization, the more likely it is that implementation will be successful; other initiatives and priorities can mitigate this effect, by subsuming attention and resources [16]. All IGNITE projects specifically reported challenges with integrating elements of their program into the EHR in settings that have many competing priorities. In the Indiana University (IU) study, the salience of implementing pharmacogenomics testing was relegated to be a lower priority issue as the health system’s priority was focused on large scale transition to a new EHR. In the Vanderbilt study, the salience of implementing the evidence was questioned. In that study, the Veteran’s Affairs (VA) system had nationally authored and proctored criteria for use of clopidogrel and alternative agents; the culture did not include routine genomic testing and the local VA hospital would consider adding routine genomic testing for clopidogrel candidate patients only if the genotyping was conducted within a clinical trial. By contrast, at all of the other sites in IGNITE, genotyping to guide the use of clopidogrel is considered standard of care; increasing genotyping for clopidogrel was considered quality improvement. Variation in the relative priority of genomics had downstream effects beyond the scope of any individual project including (1) a variety of different models being applied for data extraction and analysis and (2) informed consent processes that impacted data sharing. While most of the IGNITE sites with manually extracted data collect coded data prospectively using an informed consent model, some sites analyze existing de-identified data under a waiver of consent as non-human subjects research. Because of a policy change at NIH at the beginning of 2016, de-identified data collected with a waiver of consent may no longer be eligible for submission into the database of Genotypes and Phenotypes (dbGaP); some local IRBs may no longer certify data for dbGaP. To address this situation, dbGaP submission is done site-by-site rather than collectively across the entire IGNITE network.

#### Strategy: use data warehousing techniques

To implement specific tools within EHRs across health systems in the IGNITE network, most projects described using data warehousing techniques. The sites extract data from multiple sources to integrate clinical records across organizations into a central repository, which is one strategy to adapt and tailor innovations amongst various contexts [20]. IU’s INGENIOUS trial worked to integrate pharmacogenomics alerting and reporting into EHR and Clinical Decision Support systems at a large safety-net hospital system. However, during the course of the project the hospital system migrated from a home grown EHR to the Epic Systems Corporation electronic health record (Epic). Therefore, the IU team will be using data warehousing techniques to address the transition and migration of the recruitment and reporting. At this point, there is no standard method for creating alerts about actionable variants: each site has created its own clinical decision support (CDS) rules. Mount Sinai’s GUARDD study relied heavily on the Epic data warehouse at both Sinai and their partner network of neighborhood health centers, the Institute for Family Health, to identify potentially eligible patients and return results to providers through best practice alerts [21]. Mount Sinai has selected variants based on published guidelines from the Clinical Pharmacogenetics Implementation Consortium (CPIC) and other organizations and has prioritized those for which testing is permitted by New York State Clinical Laboratory Improvement Amendments (CLIA) regulations [22]. Because the University of Maryland (UM) project utilizes next-generation sequencing (NGS) to identify unique variants and generate highly individualized reports, they are currently providing an in person or telephone genetics consult to each patient with a positive result (pathogenic or likely pathogenic monogenic diabetes variant identified by NGS, classified for pathogenicity using the 2015 American College of Medical Genetics and Genomics/Association for Molecular Pathology (ACMG/AMP) standards as implemented in a tool they created [23, 24], and confirmed by CLIA-compliant Sanger sequencing) and uploading a copy of the laboratory report, the clinical consult note, and a genetic counseling summary letter to Epic; they also mail copies to the patient. For patients referred from outside the four study sites, these materials are sent to the referring provider via fax and to the patient by mail. Vanderbilt’s I^3^P is implementing a clinical decision support into four different EHRs in five different collaborating sites. In some systems, these data can be directly stored in the EHR in a structured format. At Vanderbilt genotype data include variants of unknown pharmacogenetics effect. These variants are stored outside the formal EHR system. If new studies suggest a variant is actionable and once CDS is built for the variant, new actionable variants can be moved into the EHR (e.g., Vanderbilt has moved into guidance and results support for tacrolimus and *CYP3A5* variants after initial launch). New CDS and actionable guidance follows oversight by a special pharmacogenetics panel of the institutional Pharmacy and Therapeutics committee. In general, guidance for specific variants has followed CPIC recommendations when these are available, and all current CDS have CPIC guidelines at this time. For other I^3^P sites, only actionable data are being transferred into the EHR. Similarly, integrating decision support into order entry was not reasonably possible at all of the I^3^P sites given their EHR systems. To compensate, it was decided to notify the provider of genomic results and implications for treatment instead by directing the information to the provider as a clinical note in the EHR that requires co-signature by the provider.

### Challenge 2: improving clinician knowledge and beliefs about genomic medicine

Knowledge by individual stakeholders about how to use innovations is important for buy-in and utilization [16]. However, for all projects, lack of provider knowledge about how to interpret genomic data and apply it to patient care was a challenge. IU indicated that this was a particularly large challenge for its project because of the variety of providers (physicians, nurse prescribers, pharmacists) across health care settings (inpatient, outpatient primary care clinics, outpatient specialty care clinics, emergency room) who might receive pharmacogenomic reports. Thus, IU conducted data analyses to understand in what settings prescriptions for the targeted medications arose and to follow patients longitudinally to evaluate what additional settings were visited, and the heterogeneity in settings [25]. For example, warfarin prescriptions were not limited to cardiology clinics, suggesting that a system-level educational effort was needed. In general, this challenge was addressed by strategies to train and educate clinicians and by disseminating materials and conducting in-person trainings.

#### Strategy: develop educational materials for clinicians

To improve clinician knowledge about why and how the genomic innovations could be used in patient care, these diverse projects all developed a broad range of educational materials to make it easier for clinicians to learn about the intervention and how to deliver it [20]. Duke provided educational materials via a website and a document on how to collect MeTree data for clinicians. Tailored clinical decision support documents were developed with stakeholder feedback to integrate just-in-time education targeted to patients and to providers. Because IU had identified challenges related to the variety of providers and settings who would interact with the pharmacogenomics program, a solution was to conduct system-wide education that required special approvals from the health care setting administrators and various educational methods (e.g., in person and web-based training): materials included PowerPoint presentations and web-based educational modules. Mount Sinai embedded resources within the Best Practice Alert that provide not only their patient’s test result, its significance and suggested actions, but also links to what genetic testing and APOL1 mean, and to low literacy materials they can print out for their patients about APOL1 and genetic risk for kidney disease. Materials developed at the University of Florida (UF) included formal continuing medical education presentations for large groups, one-on-one educational sessions for lead clinicians in target practices, quick reference guides for providers to use when results are returned, patient and provider information sheets available in print and via clinical decision support alerts in the EHR, evidence summaries for providers to use to support clinical recommendations and engage other clinicians, and a website and online genomics newsletter (http://personalizedmedicine.ufhealth.org/). UM developed information sheets for providers on when and how to make referrals and what to expect after their patients are enrolled in the study. Through I^3^P, Vanderbilt is supported by websites originally developed to support Vanderbilt’s PREDICT (germline pharmacogenomic testing) and Personalized Cancer Medicine Initiative: www.mydruggenome.org and www.mycancergenome.org, accessible publicly and for providers and staff. These websites are directly linked from the EHR systems to allow providers to learn more about actionable, relevant drug/genome interactions. MyCancer genome also includes links to relevant genomically-driven clinical trials. I^3^P found that individuals routinely believed pharmacogenomics were important, though needed decision support (provided by linking to a Web site) to be able to explain actionability of the germline or somatic variants in practice [26].

#### Strategy: conduct educational meetings with clinicians

Five projects additionally conducted educational meetings, targeted to clinicians to teach them about the clinical innovation [20]. IU presented study training programs to the health system senior leadership team, which included clinical and non-clinical administrative personnel, and training programs for the IU School of Medicine Resident and Fellow faculty leaders. IU also held in-person meetings (with repeat educational sessions due to provider turn-over), conducted journal clubs, and held a day-long continuing education (CE)/continuing medical education (CME) conference/workshop to educate clinicians about pharmacogenomics and the potential to improve patient outcomes. Mount Sinai described how they joined existing clinician meetings held at the fifteen participating clinic sites/practices to describe APOL1, genomics, and the study and answer provider questions about testing and returning results. UF planned meetings with various levels of stakeholders to ensure understanding and engagement throughout. UM conducted monthly group meetings with clinicians, divisional and departmental grand rounds, external lectures, and educational outreach visits with two genetic counselors to facilitate implementation at different sites. The Vanderbilt study team met with providers, pathologists, and IT representatives in person on location at I^3^P sites as well as through virtual meetings to educate and facilitate implementation by having a discussion to bring new ideas to the table, learn about new hurdles and try to come up with solutions.

#### Strategy: conduct educational outreach in clinical settings

Four of the projects reported strategies to conduct educational outreach, which consists of having a trained person meet with providers in their clinical setting to educate them or to provide clinical recommendations via consultation, with the intent of influencing clinical decision making [20]. IU utilized their clinician co-investigators to meet with targeted clinical specialty service/department during staff meetings to discuss study implementation and answer questions pertaining to the study. In addition, to address clinician questions regarding patients with actionable genetic variants, IU established two committees (pharmacogenomics adjudication and clinical consultation) that review PGx reports and proactively contact prescribing providers to review results and offer formal clinical consults should they be requested. IU also embarked on CE for pharmacists and nurses via an in-person conference, web-based learning modules, and journal club presentations. Content included case studies to illustrate the role of these providers in pharmacogenetics-related patient education. UF provided a range of CE programs for inter-professional clinicians and trainees, including online and live 1-h CE programs for physicians, pharmacists, nurses; an interprofessional elective course for health science center students; and a live 2-days certificate-training program for pharmacists. UF educational programs included the opportunity for participant genotyping and the ability for participants to use their personal genetic information in patient cases and other assignments to further familiarize providers with the clinical applications of genomic data in a real-world environment. UM used two genetic counselors to facilitate implementation at different study sites through in-person and telephone meetings with local study coordinators and genetic counselors. The PI has also spoken about monogenic diabetes at several different Departmental Grand Rounds and meets monthly by teleconference with personnel from all study sites. As a delivery node under the I^3^P umbrella (Vanderbilt), the University of South Dakota and Sanford School of Medicine engaged providers through a huge multi-state initiative called Imagenetics, merging Internal Medicine and Genetics to deploy translational genomics in the context of primary care. Coincident with the expansion of laboratory capability (in the area of molecular diagnostics) and informatics infrastructure (in the area of automated decision support), the University of South Dakota initiated a monthly series of *Point-Counterpoint* manuscripts exploring the pros and cons of gene based drug dosing — for all actionable CPIC gene-drug relationships — in a journal called *South Dakota Medicine*. This series has begun a robust dialogue among providers of all specialties across the entire state. To further facilitate the process of educating providers, Sanford Imagenetics has allocated additional funding to embed genetic counselors in outpatient internal medicine clinics at all major division sites across the upper Midwest.

### Challenge 3: engaging patients to participate in genomic medicine projects

One core component of an implementation process is attracting and involving appropriate stakeholders in the implementation and use of the innovation [16]. All projects faced challenges with finding providers and patients willing to enroll and, for some sites, to know at which level to initiate strategies to engage stakeholders. For most sites directed by the Vanderbilt I^3^P team, gene-based drug dosing was deployed within the context of routine clinical care; therefore, no direct patient consent was required. For example, Imagenetics at Sanford Health embeds genetic counselors in outpatient internal medicine clinics at all major division sites across the upper Midwest, facilitating rapid access to genetic counselors for providers to better identify which patients are most appropriate for pre-emptive pharmacogenetics testing and help for patients to better understand the clinical impact of their pharmacogenetics test results as their healthcare unfolds [27]. This allows early engagement with patients in their own healthcare as well as providing opportunities to describe how genomic medicine projects could be beneficial to them. However, one of Vanderbilt’s sites, the VA Tennessee Valley Healthcare System, did have to directly consent patients for enrollment, with patients explicitly consenting to sharing their data on dbGaP, rendering it more challenging to engage patients at that site. This was due to the VA national policy on data release, and there is ongoing national policy discussion about changing the data sharing and release policies of VA.

#### Strategy: use mass media to educate potential users

All six projects have used mass media, a strategy to spread the word about the innovation by communicating to large audiences [20]. Duke promoted news articles on TV, the radio, and local newspapers. The IU School of Medicine, in collaboration with the IU Kelley School of Business developed a detailed launch plan for the INGENIOUS study that included a plan for widely publicizing the study within the health system and externally to the general public (local and national) via email press releases, forwarded by senior leaders, and to local and national media by communications departments, resulting in publicity by over 207 websites and journals with a potential reader reach of over 20 million visitors per day. In addition, the Chief Medical Officer at the health system highlighted the value of the IGNENIOUS study in one of his monthly newsletters. UM sought opportunities for patient advocates to speak publicly and for news reports [28]. UF has developed a robust online presence, including its SNP•its publication, which is partnered with the Pharmacogenomics Knowledgebase, UF Health Personalized Medicine Program Twitter account (@UFPersonalMed), and development of a recurring Personalized Medicine Column in a nationally distributed electronic physician newsletter. In addition, a patient who receiving genotype-guided drug therapy at UF has been featured on a local news station and both pharmacy and local magazines and their pharmacist training programs have been featured in national pharmacy publications. Vanderbilt and Sanford Health have both also leveraged media via press and in promotional materials to promulgate use of genomics to improve care.

#### Strategy: involve patients in implementation activities

Three projects additionally involved patients in implementation activities. IU conducted a focus panel with patients to obtain their input in the creation of a pharmacogenomics patient education tool, using interactive methods from design research to assist participants in drawing on their lived experiences to explore study issues and envision solutions to improve patient recruitment for the pharmacogenetics study. This resulted in patient-authored similes to be used during recruitment, for example, “Pharmacogenetics is kind of like being able to try on clothes before you buy them instead of crossing your fingers and hoping they fit,” as well as a visual tool to aid research assistants in recruiting patients and a companion piece for patients to take home containing the most important information as determined by the session participants. Another project, (Mount Sinai), relied on a genomics community board of patients, advocates and clinicians to develop all patient and clinician-facing materials, and all implementation strategies [29]. Together, they conducted a formative study prior to the randomized trial to help guide implementation. UM developed a relationship with a patient advocate recently diagnosed with monogenic diabetes, formed an alliance to increase patient and provider awareness (http://medschool.umaryland.edu/endocrinology/mdrap.asp), and used community participatory approach to develop patient educational materials (e.g. photo novella), engagement of media, and patient public speaking opportunities.

#### Strategy: prepare patients to be active participants

In addition to increasing visibility via mass media, four projects used more personalized approaches to engage patients, preparing them to be active participants in their care, including training them to ask questions about evidence behind clinical decisions [20]. Duke prepared patients to be active participants by providing education materials and generating tailored risk reports explaining patients’ risks, why they were at risk, and steps they could take to reduce their risk. Pros and cons of the recommendations were described to facilitate discussions and shared decision making with their doctors. Mount Sinai also used this strategy by meeting with numerous patients tested for APOL1 to develop and revise an education booklet about genetic testing, managing their high blood pressure and asking their doctor about tests for kidney function. UM facilitated the insurance appeals process for monogenic diabetes genetic testing by developing a template appeal letter, conducting a systematic website analysis on monogenic diabetes to identify high quality, patient-friendly online educational resources, and providing each patient diagnosed with monogenic diabetes with a customized consult note, patient friendly letter and lab report explaining the diagnosis and its implications. The PI also led a roundtable discussion on monogenic diabetes at a consumer oriented American Diabetes Association (ADA) summit in Baltimore, providing patients and ADA program staff with opportunities to gain awareness of monogenic diabetes through informal conversation with the PI, co-I, and their patient advocate partner. Vanderbilt has made genomic testing part of the patient portal with patient-friendly explanations of genetic test results. Additionally, patients have access to examples of CDS for genetic tests at Vanderbilt through the clinical decision support knowledgebase (CDS-KB) website (www.cdskb.org). With these resources, patients can better understand the role of genetic testing and be more prepared to engage with their providers about their care.

## Discussion

We identified common challenges faced by six diverse projects related to implementing genomic services into routine clinical care and strategies identified by at least half of the projects to overcome the challenges. We describe cross-project comparisons regarding which implementation strategies were employed using a published implementation framework and typology; these strategies, can be translated to other projects and settings. These findings complement work of other NHGRI-funded consortia that also are working to further implementation of precision medicine into clinical care. The CSER (Clinical Sequencing and Exploratory Research) consortium [30] is focused on studying implications of clinical sequencing for clinical care. eMERGE (Electronic Medical Records and Genomics) [31] is a consortium that is focused on developing ways to best use EHRs for linking biorepository and phenotypic data within EHRs. This synthesis of IGNITE projects provides the necessary evidence to understand *how* precision medicine can be incorporated innovatively into practice settings and thus allow for the ongoing development of evidence about what works under which conditions in real-world settings-evidence that is not possible to obtain prior to implementation [7].

All IGNITE projects noted challenges regarding integration of specific genomic innovations into some, though not all, health system EHRs. This challenge is particularly important, as EHRs are the foundation and central point that merges genomic data and the point of care contact with the patient. Without the EHR providing the network for integrating genomic and clinical data in different forms and from different sources to provide decision support in real time, successful implementation of clinically actionable genomic data would not be possible [32]. An advantage of integrating genomic information into EHRs is improved access to data across networks, a strategy not possible with paper-based systems. However, to realize the potential of genomic implementation, there are data challenges that need to be addressed, including integration of clinical decision support systems into current workflows [33]. eMERGE found that larger sample sizes from cross-site pooling of data were important for discoveries of new genotype-phenotype associations and reuse of data for different phenotypes not included in the primary research plan; to share data in this way, the EHR algorithms were developed to be portable across sites with different systems and settings [34]. Regardless of which technology is used to link data, collaboration between research and practice is key. Precision medicine is poised to produce massive amounts of data, and one expert has described the more immediate data challenge as that of a “complex” rather than “big” data problem, with the need for solutions to link and integrate data from various sources [35]. It is interesting to consider that the implementation strategies used by projects to bring genomic data to point of care and facilitate clinical decision making could have an added benefit of feeding back outcome data to, in turn, inform health system improvement, a hallmark of a learning healthcare system [15].

Provider knowledge and beliefs about genomics influences valuation and thus uptake of new genomic services, though the gap in education is not due to lack of interest [11]. Aside from formal education, strategies can be implemented at point-of-care to educate providers about how to apply genomic data, an essential part of bringing genomic data into practice. CSER and eMERGE investigators, in a cross-network collaboration, agreed on the importance of CDS for effectively using genetic information in practice and recommended linking genetic information in an EHR to knowledge bases that put the variant into clinical context, for example by describing clinical use and actionability [36]. eMERGE and CESR investigators also ranked having access to external CDS as a priority, though as with EHR systems, it can be difficult to share CDS across different EHR products or health systems. Peterson et al. (2016) found that clinicians do heed pharmacogenomic advice delivered by decision support system, though actions taken vary by provider, underscoring the utility of meetings and outreach, as conducted by IGNITE projects, to facilitate consistent use of these systems [37]. It is important to note that among IGNITE projects, education activities were conducted not only in academic centers but also in community settings, which serve greater numbers of patients and often have less educational support. Further, offering CME credit for participation in these activities as some projects did can be important for boosting participation, as providers have many priorities and limited time for CE [38]. It is possible that deficits in clinician knowledge coupled with EHR issues could only serve to magnify the impact of individual challenges, for example, by having to educate not only about content (genetics), but also the platform (new alerts in EHR). Timing of strategies to address the combined effect of these challenges would be important, such as ensuring that EHR is functioning before educating providers so that the system will be able fully answer their questions.

IGNITE projects conducted activities to educate not only providers but also patients in order to facilitate implementation. Personalized medicine is essentially individualized even when applied on a public health scale, and thus it is important to involve patients in the implementation process to be able to take into account social norms, culture, risk perception, and family factors among other personal information. It is also necessary to engage patients to facilitate within-family communication, not only to obtain family health history information, but also pursue cascade testing. Here also implementation strategies can interact in that providers who know how to use and interpret genomic medicine innovations will be in a better position to engage patients to elicit these contextual factors that are important for a comprehensive consideration of options for and impact of genomic approaches. The Genetic Counseling Working Group of the CSER consortium conducted an analysis of all CSER projects to understand best practices for obtaining informed consent from and returning results to patients when conducting clinical sequencing. Lessons learned include importance of managing expectations in pre-test and post-test counseling that negative findings do not mean that the condition is not genetic; that both healthy and ill patients need follow-up for recommendations of incidental findings; that providers should consider individual patients’ thoughts and emotions around sequencing; and, to consider variability in reactions to genetic sequencing within a family [39]. Training providers and other allied health professionals in patient-centered approaches and advanced communication skills will be necessary to engage patients in decision-making around using genomic data [40]. MedSeq is one project that is studying how prepared physicians are to return results of pathogenic variants, likely pathogenic variants, and suspicious variants of uncertain significance to patients [41].

Comparison of these findings with other studies that have used the CFIR to identify implementation challenges underscores the notion that key constructs will vary by type of intervention and, possibly, setting. For example, as with this study, “engaging stakeholders” was identified as a distinguishing construct for a VA-based lifestyle coaching health promotion program, with a strong correlation to program referral rates; however, “networks and communication,” which refers to nature and quality of formal and informal interaction within an organization, was also identified as distinguishing for that program as well as for another on-site group weight management within the VA health system, though this construct did not stand out for IGNITE projects. The IGNITE projects could have proactively facilitated development of social networks and communication channels with the outreach and education that was necessary to educate and engage stakeholders about value of genomic services. Thus, reducing the CFIR to fewer constructs that have been shown to differentiate implementation outcomes for the sake of parsimony, as recommended by Varsi et al., may, instead, limit the frame, precluding identification of factors that may matter for some [42]. As Kirk et al. suggest based on results from their systematic review of CFIR in empirical research, it will be important for future work on implementation of any program, including those related to genomic medicine, to articulate a rationale for selecting CFIR constructs and design studies to assess relationship between implementation constructs and outcomes [17]. This work will serve to build our understanding of which factors matter in what circumstances and can be used to refine implementation strategies to promote use of genomic services more widely and contribute to the field of implementation research.

Because this study is exploratory, we are limited to hypotheses generation about which factors are important for implementing genomic medicine innovations into clinical care. Regardless, this case series is a first step in identifying challenges and strategies important for implementing genomic medicine innovations across a range of innovation types and settings. This information could be used to guide development of future endeavors to design and evaluate implementation strategies, such as identifying how strategies relate to outcomes and conducting deeper evaluations of local environments and implementation adaptations.

## Conclusions

This cross-project, cross-setting synthesis represents a collaboration by members of a network devoted to generating evidence to facilitate implementation of genomic medicine into routine clinical care. Others can learn from these experiences about challenges they might expect to encounter, namely having to work around other health system priorities for the EHR and educating clinicians and patients to facilitate their engagement with genomic services and skill in shared decision-making. Policy implications can include support to accelerate linking data across systems and broader-based education of providers and the public about how to use genomic information to make personal health decisions.

## Abbreviations

ACMG/AMP: American College of Medical Genetics and Genomics/Association for Molecular Pathology
ADA: American Diabetes Association
CDS: Clinical decision support
CDS-KB: Clinical decision support knowledgebase
CE: Continuing education
CFIR: Consolidated framework for implementation research
CLIA: Clinical laboratory improvement amendments
CME: Continuing medical education
CPIC: Clinical pharmacogenetics implementation consortium
CSER: Clinical sequencing and exploratory research
dbGaP: database of genotypes and phenotypes
EHR: Electronic health record
eMERGE: Electronic Medical Records and Genomics Network
Epic: Epic Systems Corporation
ERIC: Expert Recommendations for Implementing Change
IGNITE: Implementing Genomics in Practice
IU: Indiana University
NGS: Next-generation sequencing
NHGRI: National Human Genome Research Institute
UF: University of Florida
UM: University of Maryland
VA: U.S. Department of Veterans Affairs
